# Digital reporting in a decentralized public health system: lessons from Indonesia’s micro PPKM experience

**DOI:** 10.3389/fpubh.2026.1800888

**Published:** 2026-06-08

**Authors:** Dewi Nur Aisyah, Thifal Kiasatina, Agus Heri Setiawan, Indira Rezki Wahyuni, Alfiano Fawwaz Lokopessy, Wiku Adisasmito, Zisis Kozlakidis

**Affiliations:** 1 UCL Institute of Health Informatics, University College London, London, United Kingdom; 2 Indonesia National Task Force for the Acceleration of COVID-19 Mitigation, Graha BNPB - Jalan Pramuka Kav, East Jakarta, Indonesia; 3 Department of Public Health, Monash University Indonesia, Green Office Park BSD City, Tangerang, Banten, Indonesia; 4 Indonesia One Health University Network, FKM UI, Depok, Jawa Barat, Indonesia; 5 Faculty of Public Health, Universitas Indonesia (FKM UI), Depok, Jawa Barat, Indonesia; 6 International Agency for Research on Cancer World Health Organization, Lyon, France

**Keywords:** COVID-19, digital health, micro PPKM, pandemic strategy, public health informatics, public health policy

## Abstract

**Background:**

During the COVID-19 pandemic, Indonesia implemented Micro PPKM (Community Activity Restrictions) as a decentralized public health response, coordinated through village-level command posts. To support nationwide implementation across diverse administrative levels, a digital reporting system was developed to capture operational activities from local command posts in near real-time. As COVID-19 has transitioned from an acute emergency to longer-term disease management, this experience offers an opportunity to examine what large-scale digital reporting systems can contribute to future pandemic preparedness in decentralized low- and middle-income country (LMIC) settings. In particular, empirical evidence remains limited on how such systems can support data visibility, organizational coordination, and policy implementation across fragmented administrative structures.

**Methods:**

This study analyzed secondary data generated by Indonesia’s Micro PPKM digital reporting system between June 2021 and February 2022. Reports were submitted by authorized command post personnel—primarily public order forces and registered community volunteers—across all provinces of the country. Descriptive analyses were conducted to examine the volume, geographic coverage, actor composition, activity types, and temporal trends of reported Micro PPKM activities at national and subnational levels.

**Results:**

A total of 151,558,372 activity reports were recorded from 44,770 villages, 5,502 sub-districts, 474 districts/cities, and 34 provinces. Reporting activities spanned multiple operational domains, including public health communication, mobility restrictions, enforcement actions, and support for vaccination programs. Participation involved a combination of formal state actors and community-based contributors, reflecting a hybrid governance structure. Temporal analyses showed substantial variation in reporting intensity across activity types and policy phases, while population-adjusted comparisons revealed marked subnational heterogeneity.

**Conclusion:**

The findings demonstrate the feasibility of deploying a large-scale, digitally mediated reporting infrastructure embedded within local implementation structures to support decentralized public health reporting and operation coordination during emergencies. Beyond pandemic response, such systems offer insights into how digital tools can support data visibility and standardized reporting across decentralized public health systems. Considerations related to data quality, equity, and ethical governance remain critical for sustaining and institutionalizing similar digital public health infrastructures.

## 1 Introduction

The COVID-19 pandemic posed unprecedented challenges to public health systems worldwide, exposing limitations in state capacity, governance coordination, and the translation of national policies into effective local action (1). While the acute emergency phase of the pandemic has passed, SARS-CoV-2 continues to circulate globally and remains integrated into routine respiratory disease surveillance systems, with periodic resurgences and evolving variants reported across regions (2). As of 2025–2026, cumulative global COVID-19 cases have exceeded 770 million, and officially reported deaths have surpassed 7 million. These figures likely underestimate the true burden due to reduced testing, reporting fatigue, and changes in national surveillance practices (2, 3). This transition from acute emergency response to longer-term disease management highlights the continued importance of public health systems that can support coordination, localized decision-making, and sustained preparedness for future outbreaks. Emerging evidence also suggests that COVID-19 may contribute to longer-term population health consequences beyond the acute infection phase, reinforcing the importance of sustained surveillance and public health reporting systems for monitoring evolving health burdens over time (4).

In Indonesia, early analyses of the outbreak revealed substantial spatial and temporal heterogeneity in infection patterns across provinces, reflecting differences in population density, mobility, and local capacity (5). Cumulative confirmed COVID-19 cases in Indonesia surpassed 6.8 million, with more than 162,000 reported deaths, reflecting the sustained public health and governance challenges faced by large, decentralized low- and middle-income countries (LMICs) even beyond the peak of the pandemic (3, 6). These conditions underscore the continuing relevance of public health governance mechanisms that can support localized decision-making, coordination, and preparedness in the post-emergency phase, particularly in settings characterized by complex administrative structures, multi-level governance, and uneven system capacity.

Digital health technologies have played a central role in pandemic response efforts, enabling surveillance, contact tracing, health protocol monitoring, and real-time data integration to support public health decision-making (7, 8). Evidence from several countries suggests that digitally enabled responses—when embedded within broader governance frameworks—can enhance coordination and responsiveness during public health emergencies (9–13). However, much of the existing literature focuses on high-income or city-state contexts, where digital infrastructure tends to be more uniform and administrative systems are less fragmented, facilitating coordinated implementation (14). Emerging studies from more decentralized or resource-constrained contexts highlight a different set of challenges, including coordination gaps, uneven data completeness, and variation in local implementation capacity (15). For example, Amarakoon et al. (16) describe how reporting systems in Sri Lanka and Rwanda were shaped by infrastructural and institutional constraints, while studies in Norway emphasize the role of community participation and trust in supporting reporting and contact tracing processes (17).

These differences suggest that digital public health reporting systems are not uniform in their design or function but are shaped by underlying governance arrangements and actor configurations. While some models emphasize community-based and participatory reporting, others rely on state-mediated reporting through authorized institutional actors. Consequently, there remains limited empirical evidence on how digital public health systems operate in LMICs characterized by fragmented geographies, decentralized governance, and substantial digital divides (18).

Indonesia represents a particularly instructive case for examining these dynamics. As the world’s fourth most populous country, Indonesia is administratively divided into 34 provinces, 514 districts or cities, more than 7,000 sub-districts, and over 80,000 villages, with additional governance structures at the neighbourhood (RT/RW) level (19). The country’s legal and institutional framework recognizes pandemics and disease outbreaks as complex disasters requiring multisectoral coordination, as articulated in Law no. 24 of 2007 on Disaster Management (20). In response to COVID-19, the Indonesian government established a National Task Force for the Acceleration of COVID-19 Mitigation, led by the National Disaster Management Agency (BNPB), to coordinate cross-sectoral actions at national and subnational levels (21).

Throughout the pandemic, Indonesia implemented a series of policy measures ranging from large-scale social restrictions (Pembatasan Sosial Berskala Besar, PSBB) to progressively localized mobility controls. In early 2021, these efforts culminated in the introduction of micro-scale activity restrictions (Pemberlakuan Pembatasan Kegiatan Masyarakat Mikro, Micro PPKM), a policy explicitly designed to operationalize pandemic control at the community level (22). Central to this approach was the establishment of COVID-19 command posts (Posko COVID-19) at the village and neighbourhood levels, bringing together local government officials, healthcare workers, security forces, community leaders, and volunteers to implement prevention, control, and support activities in line with national directives. These command posts functioned as localized coordination units responsible for monitoring community mobility, enforcing health protocols, supporting testing and vaccination activities, and reporting daily operational data to higher administrative levels.

To support the monitoring and coordination of Micro PPKM, Indonesia developed and deployed a nationwide digital platform known as Bersatu Lawan COVID-19 (BLC). Initially designed to integrate surveillance data and monitor health protocol compliance, the BLC system evolved rapidly into a comprehensive digital reporting infrastructure capable of capturing aggregated, geo-referenced data on community-level public health actions in real time (23, 24). Reports submitted through the platform documented a wide range of activities, including health protocol education, mask distribution, vaccination support, and mobility supervision, enabling continuous feedback between local implementation units and central authorities. Reporting was conducted by authorized personnel at local COVID-19 command posts using standardized digital forms, with data submitted through a centralized interface and aggregated across administrative levels.

Beyond its technical function, the BLC system illustrated how digital health tools can support standardized reporting, operational coordination, and administrative visibility within decentralized LMIC settings. By standardizing reporting processes and enabling tiered data access across institutions, the platform reduced information asymmetries, enabled more standardized reporting across institutions and improved visibility of locally implemented activities within a national reporting framework. At the same time, reliance on digital reporting raised critical questions regarding equity, access, and ethical governance, particularly in contexts marked by uneven connectivity and varying local health delivery capacities.

This study examines how Indonesia operationalized Micro PPKM through the Bersatu Lawan COVID-19 (BLC) digital reporting system and documents the reporting architecture, actor configuration, geographic coverage, and reporting dynamics that emerged within a highly decentralized administrative context. Rather than evaluating policy effectiveness, the paper treats the BLC-enabled Micro PPKM experience as a national case study of digitally mediated public health implementation during crisis response. By doing so, it highlights lessons relevant to future pandemic preparedness in LMICs, particularly regarding standardized reporting design, tiered institutional access, hybrid state–community implementation arrangements, and the equity challenges created by uneven local capacity and digital infrastructure.

The significance of this dataset lies not simply in its size, but in what it captures. Unlike routine epidemiological surveillance data, the BLC Micro PPKM system recorded operational public health actions undertaken at the local level, including education, enforcement, mobility supervision, and vaccination support. This makes the dataset analytically valuable as a nationwide administrative record of how a decentralized public health response was rendered visible through standardized digital reporting across villages, sub-districts, districts, and provinces. This enables examination of reporting architecture, actor configuration, and spatial–temporal variation in implementation, which are often difficult to observe systematically in large and geographically diverse LMIC settings. These insights offer implications for the design of scalable and context-sensitive digital public health infrastructures to support future health emergency preparedness and response in decentralized settings.

## 2 Materials and methods

### 2.1 Study design

This study employed a descriptive national case study design using aggregated administrative data generated through Indonesia’s national digital public health reporting system, Bersatu Lawan COVID-19 (BLC). The analysis focused on how Micro PPKM (Pemberlakuan Pembatasan Kegiatan Masyarakat Mikro) activities were rendered visible through standardized digital reporting across multiple administrative levels within a decentralized response system. The Micro PPKM activities conducted at the village and neighbourhood levels, including health protocol education and socialization, enforcement measures, mobility supervision, testing and contact tracing support, vaccination activities, community monitoring, and distribution of logistics and social assistance.

In this paper, implementation refers to the documented reporting of locally conducted Micro PPKM activities through the BLC platform, including their volume, distribution, actor composition, and temporal dynamics. Governance is used in a narrower institutional sense to refer to the formal reporting architecture of the system, including authorized reporting roles, tiered access arrangements, and the standardized structure through which local activities were recorded and aggregated.

The study did not assess coordination effectiveness, decision quality, epidemiological impact, or the broader political processes of governance. Instead, it provides a descriptive account of how a large-scale digital reporting system functioned as an administrative infrastructure for documenting decentralized public health response.

### 2.2 Data source and reporting actors

Data was obtained from the BLC digital reporting platform, which was developed and managed by the National Task Force for the Acceleration of COVID-19 Mitigation as part of Indonesia’s integrated COVID-19 response infrastructure. The platform enabled standardized, real-time reporting of Micro PPKM activities conducted at the village and neighbourhood levels.

Digital reports were submitted by authorized personnel assigned to COVID-19 command posts, primarily consisting of:

1. Indonesian National Armed Forces (Tentara Nasional Indonesia, TNI) personnel deployed at the village level;
2. Indonesian National Police (Kepolisian Negara Republik Indonesia, POLRI) personnel responsible for public order and mobility supervision; and
3. Registered community volunteers, including Behavioral Change Ambassadors, who supported implementation and reporting activities.

Access to the BLC system required institutional authentication, and reporting privileges were restricted to designated personnel. Members of the general public did not submit reports directly through the platform.

### 2.3 Data collection procedures

Micro PPKM activities at the local level were documented through standardized digital reporting forms embedded within the BLC platform. In this study, an activity report refers to a single digital submission documenting one reported operational activity conducted by a COVID-19 command post. Depending on local implementation practices, multiple activity reports could be submitted by the same command post within a single day. Repeated submissions from the same command post were possible and could legitimately reflect multiple operational activities conducted across different times or locations within a reporting period. Each submission was accompanied by an automated timestamp and GPS-based location recording at the point of reporting, enabling activities to be documented in near real time. As such, repeated submissions did not necessarily represent duplicate reports but rather reflected the operational nature of Micro PPKM implementation activities conducted by authorized personnel across decentralized settings.

The reporting was conducted by authorized personnel at COVID-19 command posts operating at village and neighbourhood levels across Indonesia. Reporting was carried out on an ongoing basis as part of daily operational activities during the Micro PPKM implementation period, with designated personnel responsible for documenting and submitting activity reports through the Bersatu Lawan COVID-19 (BLC) platform.

Data were submitted using a structured digital reporting form embedded within the BLC system. Each report was completed by authorized users and transmitted in near real time to a centralized system, where it became accessible through a national dashboard for monitoring and coordination by authorized national and subnational authorities.

The reporting form included standardized and mandatory fields covering the type of activity conducted, location, time of implementation, and supporting metadata. Activity categories were predefined within the BLC system and selected by reporting personnel from standardized options, rather than being entered as free-text responses. Reported activities included, for example, health protocol education and socialization, enforcement measures, mobility supervision, testing and contact tracing support, vaccination activities, community monitoring, and distribution of logistics and social assistance. Geographic coordinates were captured automatically through Global Positioning System (GPS) functionality, enabling spatial referencing, aggregation, and validation of reported activities.

Access to the BLC system required institutional authentication, and reporting privileges were restricted to designated personnel. Members of the general public did not submit reports. This role-based access structure enhanced accountability by ensuring that each submission could be attributed to identifiable institutional actors, while also supporting data quality through controlled reporting processes and reduced variability in data entry. Reports were linked to institutional identities, with role-based access controls governing data submission and visibility across administrative levels.

### 2.4 Study period and data scope

The analysis covered reports submitted between June 2021 and February 2022, corresponding to the period of nationwide implementation and more stable operationalization of the Micro PPKM reporting system through the Bersatu Lawan Covid-19 (BLC) platform. Although the platform had been introduced earlier in 2021, this study focused on June 2021 onward to capture the phase during which reporting structures, command post deployment, and reporting practices had become more standardized across provinces. Only reports originating from officially registered COVID-19 command posts and submitted by authorized TNI, POLRI, or registered volunteer personnel were included. Individual-level health data, personal identifiers of community members, and clinical outcomes were not collected or analyzed.

### 2.5 Data management and analysis

Data was extracted from the Bersatu Lawan COVID-19 (BLC) platform in aggregated form and accessed through a centralized dashboard system that provides real-time visualization of reported activities. Aggregated datasets could be securely exported from the dashboard for offline analysis; these datasets contained only summary counts of reported activities and did not include any individual-level or personally identifiable information. All data processing and analysis were conducted in a secure analytical environment.

The BLC platform employs a standardized digital reporting structure, in which all data fields are predefined and mandatory at the point of entry. Key variables—including personnel information (e.g., name, institutional affiliation, role), administrative location (province, district/city, subdistrict, village), activity type, and reporting timestamp—are captured through structured forms using dropdown selections rather than free-text input. In addition, geographic coordinates (GPS data) are automatically recorded at the point of reporting, enabling spatial referencing of reported activities. This system design supports administrative completeness and consistency across the reporting units, minimizes missing data within required reporting fields, and reduces the need for extensive post-extraction data cleaning. However, standardized reporting structures do not eliminate potential reporting biases, including underreporting, variation in local reporting capacity, and differences in the availability of authorized reporting personnel across regions.

Basic monitoring and consistency checks were conducted to support administrative alignment across reporting periods. These activities focused on consistency of administrative identifiers, temporal alignment of submitted reports, and routine oversight through hierarchical dashboard monitoring across administrative and institutional levels. Provincial authorities monitored reporting activity at district/city and village levels, while centralized command structures within TNI, POLRI, and the national COVID-19 task force routinely reviewed dashboard outputs to support real-time coordination and operational situational awareness during the pandemic response. These mechanisms were intended to support reporting continuity and implementation oversight rather than formal audit-level verification of each individual report.

Data extracted from the BLC system were analyzed using descriptive methods to examine: (a) geographic coverage of reporting command posts; (b) volume and temporal trends of reported activities; (c) distribution of reporting actors across institutional categories (TNI, POLRI, volunteers), and (d) typology of Micro PPKM activities. In addition to absolute activity counts, population-adjusted measures were used to assess variation in reporting intensity across provinces. An exploratory Pearson correlation analysis was additionally conducted to examine the relationship between weekly national COVID-19 incidence and weekly reported Micro PPKM activity volumes during the study period.

Analyses were conducted at national and subnational levels, with findings summarized using frequencies, proportions, temporal patterns, and exploratory correlation measures. All analyses were conducted using R. Spatial visualization was used to examine geographic patterns in reporting coverage and activity distribution across administrative levels. An example of such visualization, illustrating compliance mapping across district/city, sub-district, and village levels, is provided in Supplementary Figure S1.

### 2.6 Governance and ethical considerations

The BLC system was implemented in accordance with national data governance frameworks for public health surveillance in Indonesia, including regulations governing the use of administrative and health data for public health response and emergency management (20, 25). These frameworks emphasize data protection, restricted access, and the use of data for authorized public health purposes.

Digital reporting was conducted exclusively by authorized TNI, POLRI, and registered volunteer personnel operating under formal institutional mandates. Access to the BLC system and underlying data was granted through formal authorization by relevant national authorities responsible for COVID-19 response and digital reporting infrastructure. Data access was restricted to authorized personnel, and all data handling followed applicable data governance and security protocols.

All data analyzed in this study were aggregated and non-identifiable, and no individual-level or personal health information was accessed or extracted. As the study analyzed secondary administrative data collected for public health response and did not involve human subjects research, ethical approval was not required under applicable national regulations (26).

### 2.7 Methodological limitations

This study has several methodological limitations related to the use of aggregated administrative data and variation in reporting capacity across decentralized implementation settings. Potential reporting biases, including underreporting and differences in local reporting capacity, may have influenced the observed variation in reported activity volumes across regions. Reporting intensity was influenced by the availability and deployment of authorized reporting personnel, including TNI, POLRI, and trained volunteers, as well as by variations in digital infrastructure and local administrative capacity. Consequently, reported activity volumes should be interpreted as reflecting both implementation effort and reporting capability across regions. In addition, although the reporting system incorporated mandatory standardized fields, automated timestamps, GPS-based reporting, and hierarchical monitoring mechanisms, individual activity reports were not formally validated or audited at the record level. As reporting was conducted in real time by multiple authorized personnel across decentralized settings, some reported activities may have reflected overlapping operational activities or multiple submissions related to the same implementation event.

To support reporting consistency, the Micro PPKM system incorporated coordinated implementation mechanisms, including structured training for reporting personnel, hierarchical monitoring and supervision across administrative levels (e.g., national to provincial, provincial to district/city), and routine monitoring conducted by the national COVID-19 task force. These measures were intended to support more standardized reporting practices and reduce variability across regions, although differences in local capacity may still persist.

Additionally, as the analysis relied on aggregated administrative data, it cannot capture informal or unreported community actions, nor does it allow causal inference regarding the effectiveness of Micro PPKM interventions. However, the Micro PPKM policy was designed as a structured and centralized framework for COVID-19 response at the community level, which may have reduced the extent of informal or unreported activities by channelling implementation through formalized command post structures.

## 3 Results

The following results describe the extent to which the digital reporting system was adopted and utilized across Indonesia’s decentralized administrative structure. Rather than reflecting policy effectiveness directly, these findings provide an indication of reporting reach, system uptake, and the degree to which digital reporting became embedded within local implementation arrangements.

### 3.1 National coverage and spatial distribution of micro PPKM reporting

During the study period, 29,913 COVID-19 command posts were established and registered in the BLC Micro PPKM reporting system, equivalent to 37.17% of the 80,470 villages across Indonesia. These command posts reported Micro PPKM activities that collectively covered 44,770 villages, 5,502 sub-districts, 474 districts/cities, and all 34 provinces. The number of villages covered by reported activities was therefore higher than the number of registered command posts because a single command post could implement and report activities spanning more than one village.

**Figure 1:**
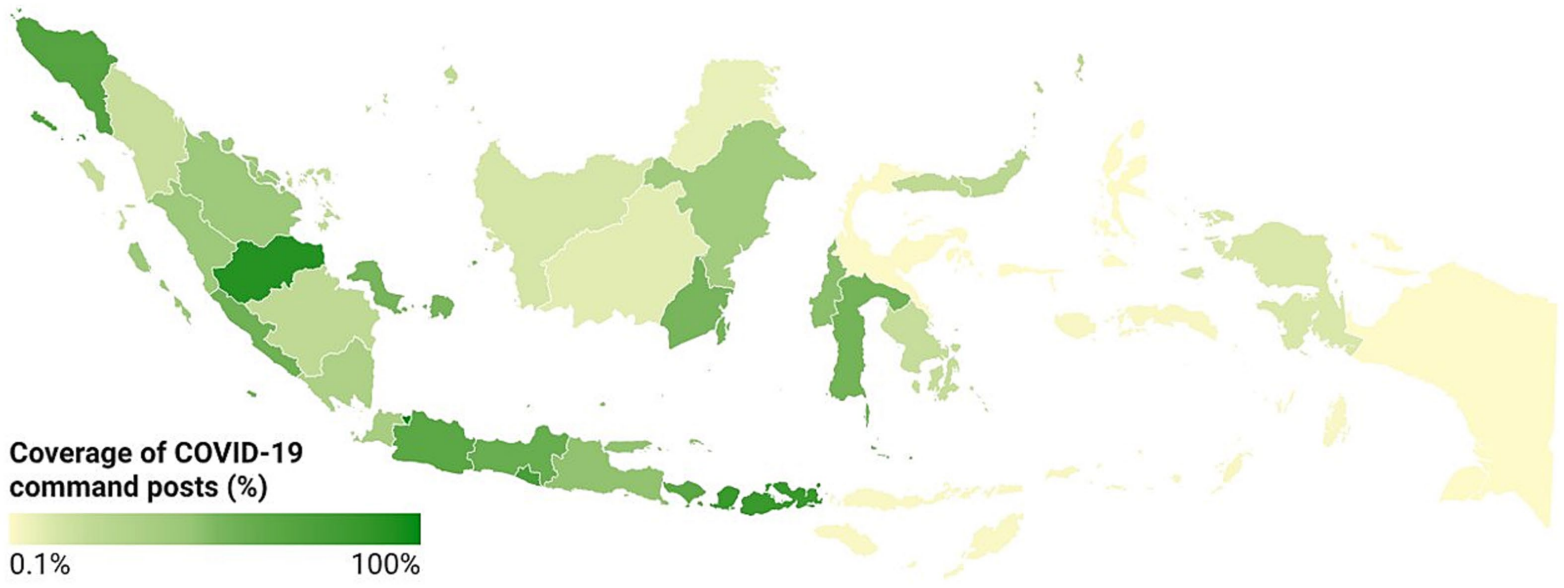
Geographic distribution of COVID-19 command post coverage across Indonesian provinces.

As shown in Figure 1, the distribution of COVID-19 command posts varied substantially across regions. Higher coverage was observed in Java and Bali, followed by Sumatra, while several provinces achieved near-complete coverage (Figure 1). In contrast, lower levels of coverage were observed in other regions, indicating uneven implementation across Indonesia’s decentralized administrative system. Detailed numerical values underlying the provincial distribution are provided in the Supplementary Table 1.

### 3.2 Leadership structure and composition of COVID-19 command posts

**Figure 2:**
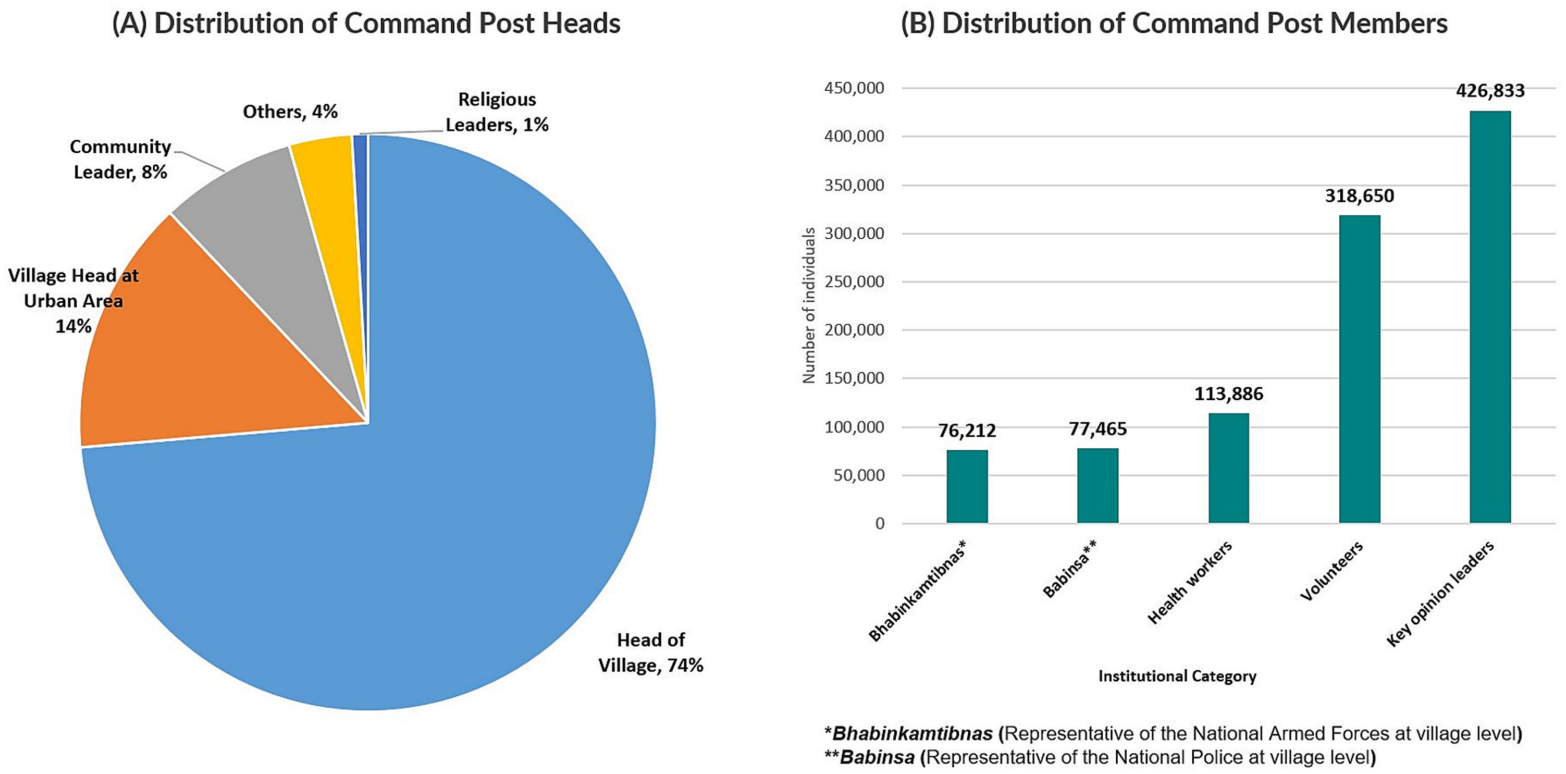
Distribution of reporting actors across institutional categories: **(A)** Distribution of command post heads by institutional affiliation and **(B)** distribution of command post members by institutional affiliation.

Figure 2 presents the distribution of leadership roles and membership composition within regional COVID-19 command posts under the Micro PPKM framework. Village heads represent the formal administrative authority at the local level and are responsible for coordinating government-led programs within their jurisdictions. Community leaders refer to locally recognized informal leaders who play a key role in mobilizing community participation, while religious leaders hold social influence and can support public health messaging and compliance within faith-based communities. The presence of these different actor groups reflects the integration of formal governance structures with community-based leadership in the implementation of Micro PPKM, which is characteristic of Indonesia’s decentralized administrative system.

As shown in the left panel of Figure 2, leadership positions were predominantly held by village heads, accounting for approximately 74% of all command post heads. This was followed by village heads in urban areas (14%), community leaders (8%), religious leaders (1%), and other actors (4%). These data indicate that formal leadership of command posts was largely embedded within existing village administrative structures.

The right panel of Figure 2 illustrates the composition of command post members across institutional and community categories. Based on the registration data collected from BLC, the total number of command post members across all 34 provinces was 1,013,046, with West Java having the most members in post (155,941 members), followed by Central Java (147,213 members), Aceh (136,581 members), East Java (135,056 members), and South Sulawesi (58,028 members). Membership included key opinion leaders (426,833) and volunteers (318,650) as the largest groups, followed by health workers (113,886). State security personnel were represented through Babinsa (TNI representatives at the village level, 77,465) and Bhabinkamtibmas (POLRI representatives at the village level, 76,212).

While command post membership comprised a diverse set of actors, digital reporting through the BLC platform was restricted to authorized personnel, namely TNI, POLRI, and registered volunteers, in accordance with institutional access controls. Other command post members, including health workers and key opinion leaders, did not submit reports directly through the system but participated in implementation and support activities at the local level.

### 3.3 Typology of micro-level public health activities

**Figure 3:**
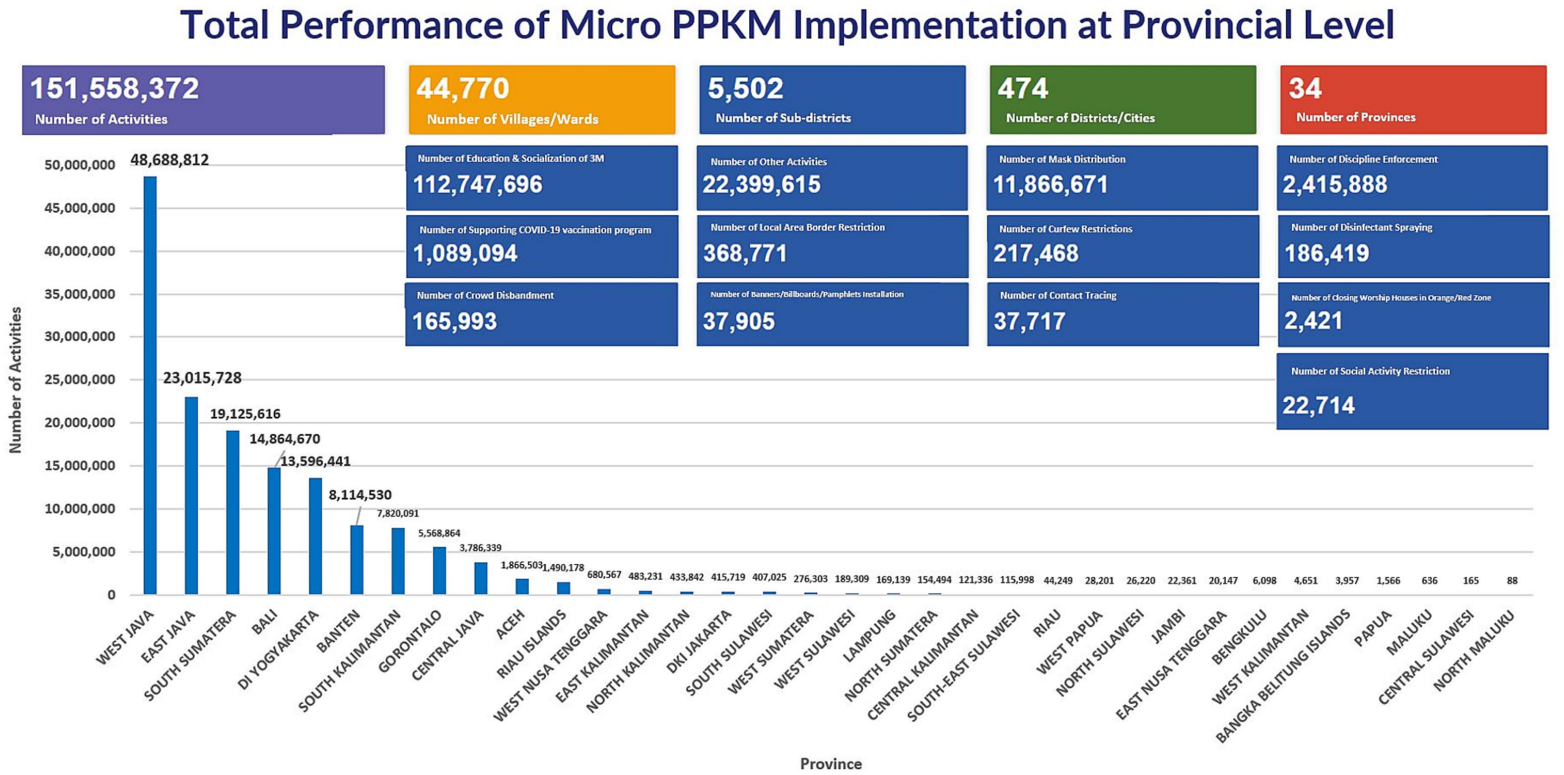
The total performance of micro PPKM implementation at the provincial level.

Figure 3 presents the aggregate volume and distribution of Micro PPKM activities reported through the BLC platform at the provincial level. During the study period, a total of 151,558,372 Micro PPKM activity reports were recorded nationwide. These report activities covered 44,770 villages, 5,502 sub-districts, 474 districts or cities, and spanning all 34 provinces.

As shown in Figure 3, health protocol education and socialization constituted the largest share of reported activities by a substantial margin. Other activities, including mask distribution and vaccination support, were reported less frequently, while more resource-intensive interventions—such as mobility control, enforcement measures, and contact tracing—accounted for a relatively small proportion of total reported activities.

The distribution of reported activities also varied markedly across provinces, with higher volumes concentrated in more populous regions, particularly on Java. In contrast, provinces in eastern Indonesia reported substantially lower activity volumes, reflecting geographic and structural variation in reporting coverage (Figure 3).

### 3.4 Temporal dynamics of reporting intensity

**Figure 4:**
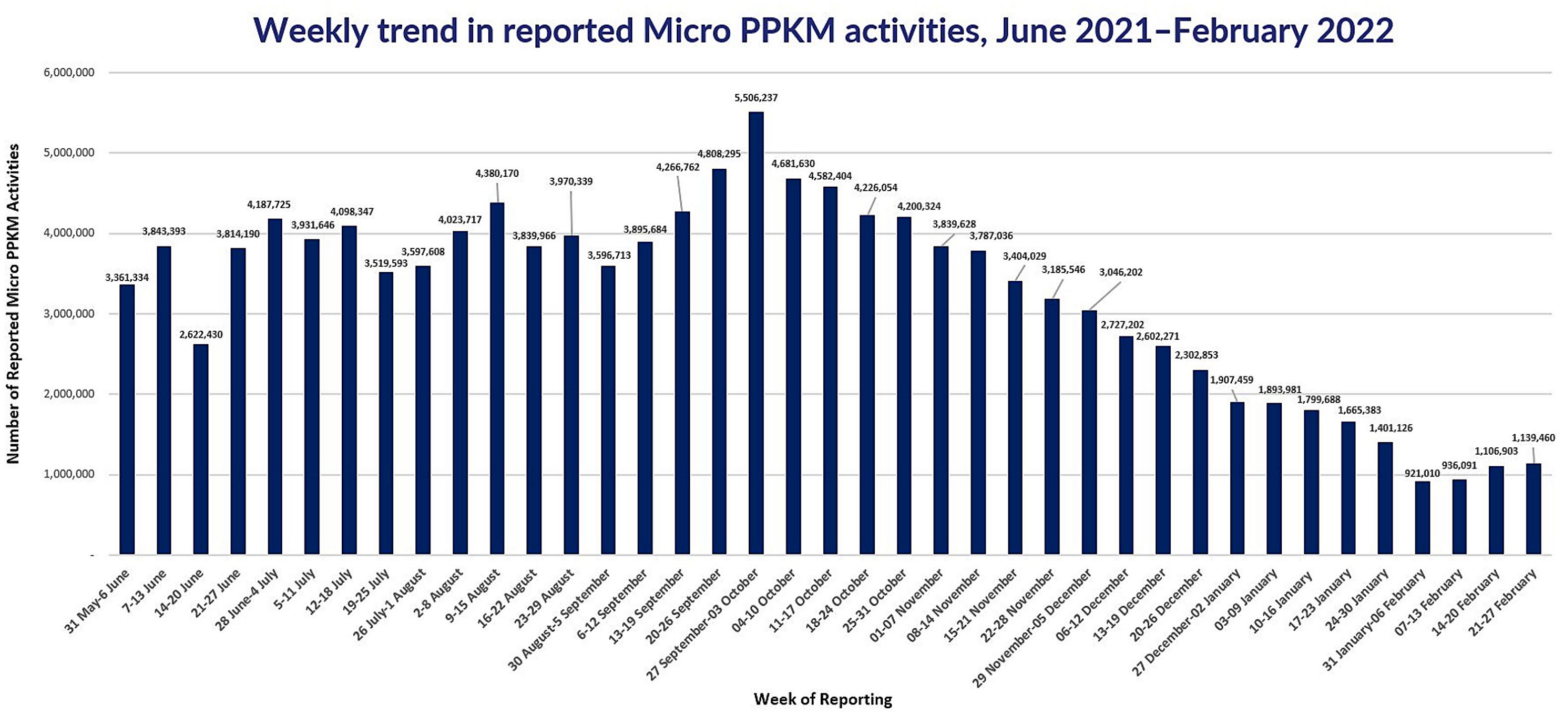
Weekly trend development of total micro PPKM performance in June 2021–February 2022.

Reporting activity through the BLC platform demonstrated clear temporal variation over the study period (Figure 4). In the early phase following the nationwide scale-up of Micro PPKM reporting during mid-2021, reporting volumes increased rapidly as local command posts were established, and reporting mechanisms became operational. Reporting activity reached its peak during periods of intensified pandemic control measures, reflecting heightened implementation of community-level interventions and increased reporting activity across regions. Following this peak, reporting volumes showed a gradual decline, corresponding with changes in pandemic conditions and adjustments in public health measures over time. Despite this decline, reporting activity remained consistently present across the study period, indicating sustained use of the BLC system as part of ongoing response and monitoring efforts.

The number of COVID-19 command post performances (program activities) continued to increase from June 2021 until early October 2021 where the weekly number activities reached 5.5 million reports at its peak as shown in Figure 4. However, along with the declining number of COVID-19 cases and the massive rollout of the COVID-19 vaccination program in Indonesia, there was decline in reporting, reflecting the progress and needs imposed by the pandemic waves. More detailed weekly trends by activity type are presented in Supplementary Figure S2.

Exploratory correlation analysis demonstrated a weak negative relationship between weekly national COVID-19 incidence and weekly reported Micro PPKM activity volumes (Pearson’s r = −0.14, *p* = 0.41). This finding suggests that reporting activity through the BLC platform did not solely mirror epidemiological trends, but also reflected broader operational, administrative, and community-level implementation activities conducted throughout different phases of the pandemic response. Temporal trends illustrating the relationship between weekly COVID-19 incidence and Micro PPKM activity reports are presented in Supplementary Figure S3.

### 3.5 Population-adjusted distribution of micro PPKM activities

**Figure 5:**
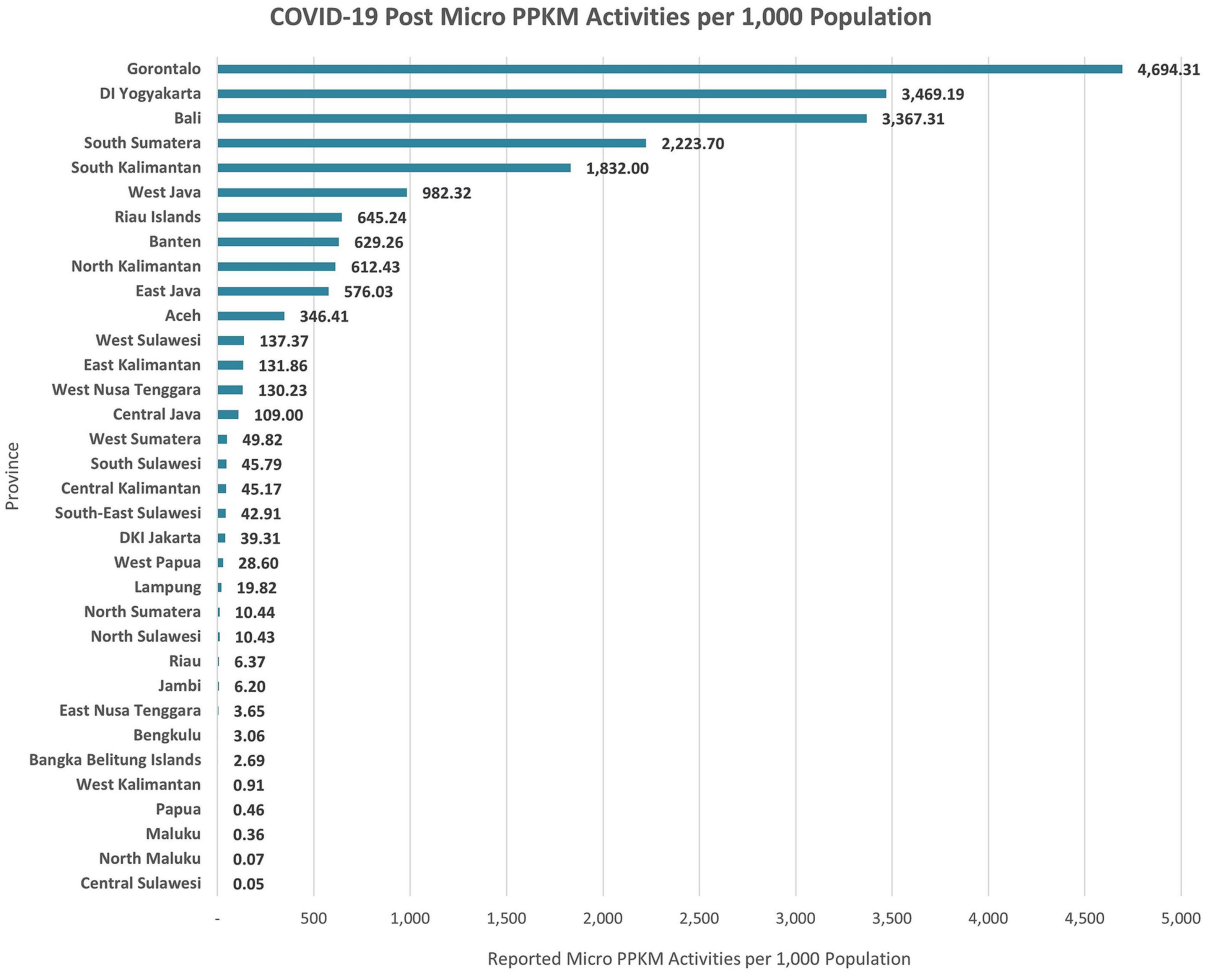
COVID-19 command post micro PPKM activities per 1,000 population.

Figure 5 presents the distribution of Micro PPKM activities normalized by population size, expressed as the number of reported activities per 1,000 population at the provincial level. This population-adjusted metric provides a complementary perspective to absolute activity counts by accounting for differences in provincial population size.

When adjusted for population size, substantial variation in reporting intensity across provinces becomes more apparent (Figure 5). Several provinces with smaller populations exhibited relatively high activity rates, while more populous provinces showed lower rates when normalized by population size. At the lower end of the distribution, some provinces reported minimal activity relative to their population, highlighting disparities in reporting intensity across regions.

## 4 Discussion

### 4.1 Digital reporting and operational coordination in a decentralized pandemic response

The findings of this study show that the Micro PPKM response generated a large-scale digitally mediated reporting system through which locally implemented activities were documented across Indonesia’s decentralized administrative structure. The BLC platform functioned as a centralized information infrastructure, integrating reports from thousands of COVID-19 command posts operating across Indonesia’s decentralized administrative system. This reflects a broader trend in which digital platforms are increasingly used to support standardized reporting and enhance administrative visibility of public health interventions during emergencies, particularly in LMICs (27, 28). Similar experiences were also reported in other COVID-19 response settings, including Taiwan, where digital technologies were used to support integrated reporting, inter-agency coordination, and operational monitoring during the pandemic response (29).

Rather than replacing existing governance arrangements, the digital reporting system was layered onto established village-level administrative structures. Such an approach aligns with prior evidence suggesting that digital health systems are more likely to function effectively when they complement existing institutional configurations rather than impose entirely new governance models (30).

The operational role of the BLC platform was also reflected in governmental implementation guidance and prior studies describing its use for monitoring Micro PPKM activities, situational coordination, and reporting oversight during the COVID-19 response in Indonesia (23, 31–33). Previous studies have additionally documented the use of the BLC system for monitoring community-level compliance with COVID-19 prevention measures, including implementation of health protocols such as mask-wearing, handwashing, and physical distancing (23). In this context, the platform functioned not only as a reporting interface, but also as part of a broader operational monitoring and coordination infrastructure supporting decentralized pandemic response.

Comparable digital COVID-19 dashboards and reporting platforms were implemented in several countries during the pandemic, including South Korea, Taiwan, Saudi Arabia, and the United Kingdom, primarily to support epidemiological surveillance, contact tracing, mobility monitoring, and public communication (8–11, 34–36). Many published systems functioned predominantly as centralized surveillance or decision-support dashboards focused on case monitoring and situational awareness.

In contrast, the Bersatu Lawan COVID-19 (BLC) system combined dashboard visualization with structured operational reporting from decentralized implementation units. Rather than focusing solely on epidemiological indicators, the system documented community-level implementation activities conducted through village-based command posts, including health protocol monitoring, vaccination support, mobility supervision, and public health outreach activities. This enabled the platform to function not only as a surveillance interface, but also as an administrative coordination and implementation monitoring infrastructure across multiple governance levels within a highly decentralized LMIC setting.

### 4.2 State-led reporting and the configuration of community participation

A central contribution of this study lies in clarifying the distinction between participation in implementation and authority in digital reporting. While COVID-19 command posts included a wide range of actors—such as volunteers, health workers, and local opinion leaders—digital reporting through the BLC platform was restricted to authorized personnel, primarily from TNI, POLRI, and registered volunteers.

This configuration challenges assumptions that digital public health systems in LMICs are inherently community-driven or citizen-led. Instead, the findings suggest a model in which state actors play a central role in supporting data standardization, accountability, and continuity, particularly during crisis response (37, 38). Community participation, in this context, was embedded primarily in implementation and support activities rather than direct data submission or integration.

This composition of leadership roles reflects the hybrid nature of local governance in Indonesia, where formal administrative authority is complemented by socially embedded actors such as community and religious leaders. Such an arrangement highlights how decentralized public health responses may rely on the interaction between formal state structures and community-based leadership, even when digital reporting authority remains institutionally controlled.

### 4.3 Understanding variation through absolute and population-adjusted measures

The inclusion of population-adjusted analysis provides additional insight into the observed heterogeneity of Micro PPKM implementation. While absolute activity volumes were concentrated in densely populated provinces, normalization by population size revealed alternative patterns of reporting intensity across regions.

This contrast highlights the importance of employing multiple analytical lenses when interpreting administrative digital health data. Previous studies have cautioned that reliance on aggregate counts alone may obscure relative intensity and distribution of interventions, particularly in geographically and demographically diverse settings (39, 40). The combined use of absolute and per capita measures therefore contributes to a more nuanced understanding of micro-level public health implementation.

### 4.4 Digital visibility, capacity constraints, and uneven representation

The observed distribution of activity types and reporting intensity provides insight into how decentralized public health responses are operationalized in practice. The predominance of health education and socialization activities reflects a pattern widely observed in public health responses, where risk communication and community engagement are prioritized due to their scalability and relatively low resource requirements. Global guidance during the COVID-19 pandemic emphasized communication strategies as a core intervention, particularly in settings with heterogeneous local capacity (41).

In contrast, activities requiring higher levels of coordination, technical expertise, or resource mobilization—such as contact tracing or enforcement-related interventions—tend to be more unevenly implemented across decentralized systems. This is consistent with broader health systems literature, which highlights how variations in workforce capacity, infrastructure, and institutional coordination shape the implementation of more complex interventions in LMIC contexts (42, 43).

Similarly, the observed variation in reporting intensity across provinces likely reflects a combination of implementation dynamics and reporting capacity. Differences in population size, administrative organization, and digital infrastructure may influence both the delivery of public health activities and the ability of local systems to generate and transmit data, particularly in decentralized settings where variation in decision space and system capacity is expected (44, 45). In particular, higher population-adjusted activity rates observed in some smaller provinces may reflect more concentrated reporting or more consistent documentation practices, while lower rates in more populous regions may indicate dilution effects or constraints in reporting capacity. These patterns highlight the importance of interpreting population-adjusted indicators within the broader context of system capacity and reporting practices in decentralized settings.

These patterns are closely linked to broader structural conditions shaping digital visibility within decentralized public health systems. Differences in reporting coverage and intensity are likely influenced by factors such as digital connectivity, geographic accessibility, and the deployment of authorized reporting personnel. As a result, digital reporting systems may simultaneously enhance visibility in some regions while rendering other areas comparatively less visible.

This pattern is consistent with existing literature on digital health gaps in LMICs, which emphasizes that digitalization can reproduce existing inequalities if underlying infrastructural and institutional disparities are not addressed (46). Consequently, data generated through digital reporting platforms should be interpreted as reflecting both implementation activity and reporting capacity.

The exploratory correlation analysis further supports this interpretation. The absence of a strong positive relationship between weekly COVID-19 incidence and Micro PPKM reporting activity suggests that the BLC system functioned beyond a purely reactive epidemiological surveillance mechanism. Reporting activity likely reflected broader operational processes, including sustained public health outreach, mobility monitoring, vaccination support, enforcement activities, and administrative coordination that continued even during periods of declining transmission. This pattern highlights how digital reporting systems embedded within decentralized governance structures may capture longer-term operational and institutional dynamics that are not solely driven by fluctuations in case incidence.

### 4.5 Ethical and policy implications of digitized public health reporting

From an ethical perspective, the findings raise questions regarding representation, inclusivity, and governance within digital public health systems. When reporting authority is limited to institutional actors, certain forms of community action may remain undocumented, while others are more readily captured within centralized dashboards. Although this study did not examine individual-level data or consent processes, it underscores the importance of transparency and accountability mechanisms in digital public health governance, especially as in this case the local community-embedded public health leadership largely coincided with the local community leadership (46, 47).

For policymakers, the Micro PPKM experience suggests that large-scale digital reporting systems can enable standardized data collection and administrative visibility across multiple administrative levels. However, sustaining such systems beyond crisis contexts may require continued investment in local capacity, interoperability, and governance frameworks to ensure equitable, expanded and effective use (48).

Importantly, the broader relevance of the Micro PPKM experience does not necessarily depend on replicating the extraordinary scale of personnel mobilization observed during the COVID-19 emergency. Rather, the transferable value of the system lies in its digital reporting architecture, standardized reporting workflows, tiered coordination mechanisms, and integration of localized implementation into a centralized monitoring framework. Similar approaches may be adapted for other public health priorities—including infectious disease surveillance, immunization outreach, environmental health monitoring, disaster preparedness, or behavioral risk surveillance—using different scales of manpower, reporting frequency, and institutional involvement depending on programmatic needs and available resources.

### 4.6 Limitations and directions for future research

Several limitations should be acknowledged. First, the study relied on aggregated administrative data and could not capture informal or unreported community activities. Second, the analysis did not assess the effectiveness of Micro PPKM interventions or their impact on epidemiological outcomes. Third, observed variation in reporting intensity may reflect differences in reporting capacity rather than implementation effort alone. Fourth, administrative reporting data are inherently limited in their interpretive scope. While such data are useful for describing reporting structures, actor configuration, and system reach, they do not capture how coordination was experienced, negotiated, or enacted in practice. In particular, this study does not assess decision-making processes, coordination dynamics, or interactions between actors at different administrative levels. As a result, the findings should be interpreted as reflecting the institutional and reporting architecture of the system, rather than the full complexity of governance processes within decentralized public health responses.

Future research could extend this work by linking digital reporting data with health outcomes, conducting qualitative studies on local reporting practices, or examining the sustainability of digital public health platforms beyond emergency response periods.

## 5 Conclusion

This study provides a descriptive account of how Indonesia operationalized Micro PPKM policy through a national digital reporting platform during COVID-19 response. Using aggregated administrative data, the analysis documents the scale, distribution, and variation of reported public health activities across a highly decentralized setting.

The findings show that large-scale digital reporting can create nationwide visibility of localized implementation activities, while also revealing substantial heterogeneity across regions, actor groups, and reporting intensity. These patterns are important not because they demonstrate coordination effectiveness directly, but because they show what kinds of reporting infrastructures, institutional arrangements, and data architectures can be deployed at scale during crisis response.

As countries move from emergency response toward longer-term preparedness planning, Indonesia’s Micro PPKM experience offers lessons for the design of future public health reporting systems in decentralized LMIC settings. These lessons include the value of standardized reporting structures, clearly defined institutional roles, and attention to equity, local capacity, and digital infrastructure when seeking to institutionalize preparedness-oriented digital systems beyond the immediate crisis period.

## Data Availability

The datasets analyzed in this study are derived from aggregated administrative records generated through Indonesia’s national COVID-19 digital reporting platform (Bersatu Lawan COVID-19). The data are not publicly available due to data governance and institutional access restrictions but may be made available from the corresponding author upon reasonable request and subject to approval by the National Agency for Disaster Countermeasure (BNPB). Requests to access these datasets should be directed to DA, dewina.aisyah14@gmail.com.
