# Supplementary figures and images for: Digital reporting in a decentralized public health system: lessons from Indonesia’s micro PPKM experience
Source: Front Public Health. 2026 Jun 8;14:1800888. doi: 10.3389/fpubh.2026.1800888 (PMC13284058; doi:10.3389/fpubh.2026.1800888)

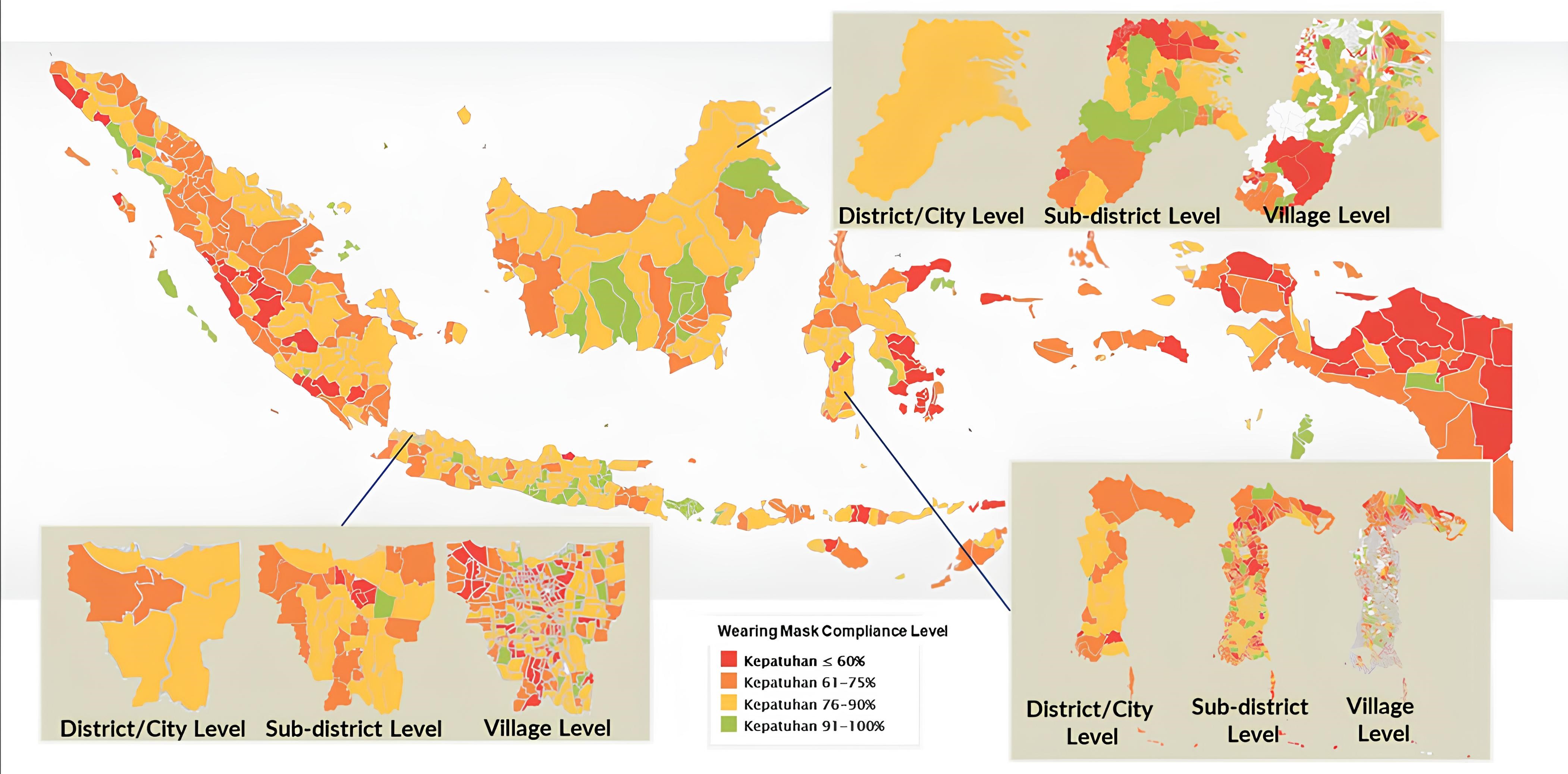

Supplement: Supplementary file 1 [file Image_1.JPEG]

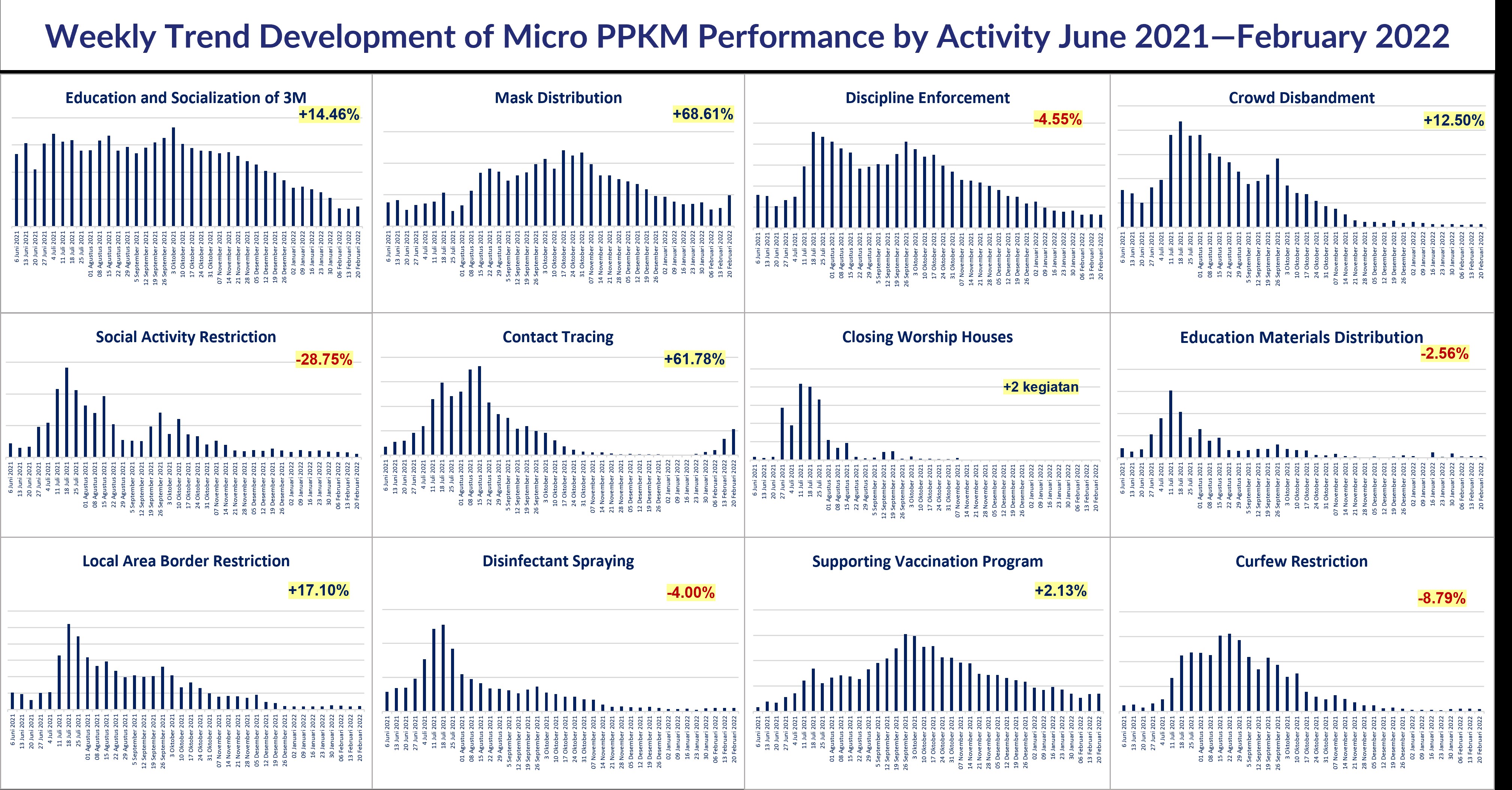

Supplement: Supplementary file 2 [file Image_2.JPEG]

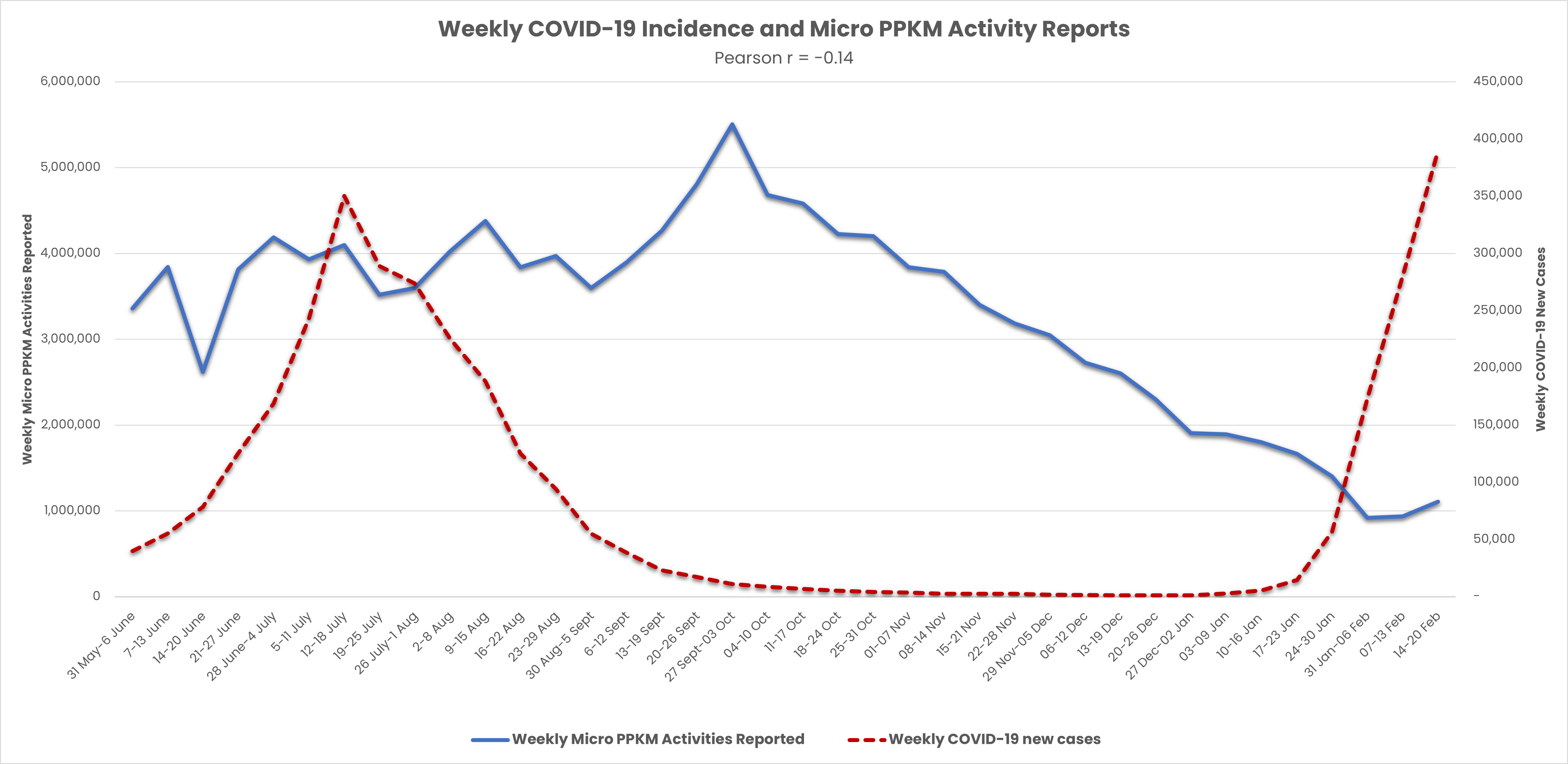

Supplement: Supplementary file 3 [file Image_3.PNG]
