# Supplementary material for: Digital reporting in a decentralized public health system: lessons from Indonesia’s micro PPKM experience
Source: Front Public Health. 2026 Jun 8;14:1800888. doi: 10.3389/fpubh.2026.1800888 (PMC13284058; doi:10.3389/fpubh.2026.1800888)
Supplement: Supplementary file 4 [file Table_1.DOCX]

Supplementary Table 1. Provincial distribution of COVID-19 command post targets, established posts, and coverage (%) in Indonesia.

| **No** | **Province** | **Target of COVID-19 posts established** | **Number of COVID-19 Command Post Established** | **% of COVID-19 command post established** |
| --- | --- | --- | --- | --- |
| 1 | DKI Jakarta | 267 | 267 | 100.0% |
| 2 | Jambi | 1,562 | 1,443 | 92.4% |
| 3 | West Nusa Tenggara | 1,063 | 891 | 83.8% |
| 4 | DI Yogyakarta | 414 | 307 | 74.2% |
| 5 | Aceh | 6,509 | 4,466 | 68.6% |
| 6 | West Java | 5,832 | 3,755 | 64.4% |
| 7 | Bali | 653 | 420 | 64.3% |
| 8 | Central Java | 8,016 | 4,648 | 58.0% |
| 9 | Bengkulu | 1,515 | 821 | 54.2% |
| 10 | South Sulawesi | 2,975 | 1,548 | 52.0% |
| 11 | Bangka Belitung Islands | 366 | 187 | 51.1% |
| 12 | South Kalimantan | 1,971 | 1,004 | 50.9% |
| 13 | West Sulawesi | 639 | 308 | 48.2% |
| 14 | East Java | 7,863 | 3,702 | 47.1% |
| 15 | Riau | 1,876 | 755 | 40.2% |
| 16 | West Sumatera | 1,160 | 466 | 40.2% |
| 17 | East Kalimantan | 1,002 | 368 | 36.7% |
| 18 | Banten | 1,502 | 505 | 33.6% |
| 19 | Lampung | 2,642 | 808 | 30.6% |
| 20 | North Sulawesi | 1,791 | 401 | 22.4% |
| 21 | South Sumatera | 3,263 | 664 | 20.3% |
| 22 | Gorontalo | 722 | 144 | 19.9% |
| 23 | Riau Islands | 395 | 77 | 19.5% |
| 24 | Southeast Sulawesi | 2,301 | 384 | 16.7% |
| 25 | North Sumatera | 6,104 | 955 | 15.6% |
| 26 | West Kalimantan | 2,073 | 241 | 11.6% |
| 27 | West Papua | 1,632 | 165 | 10.1% |
| 28 | Central Kalimantan | 1,540 | 117 | 7.6% |
| 29 | North Kalimantan | 466 | 26 | 5.6% |
| 30 | Maluku | 1,180 | 18 | 1.5% |
| 31 | East Nusa Tenggara | 3,202 | 34 | 1.1% |
| 32 | Central Sulawesi | 1,953 | 10 | 0.5% |
| 33 | Papua | 4,866 | 7 | 0.1% |
| 34 | North Maluku | 1,155 | 1 | 0.1% |
